# Wieviel Digitalisierung braucht die HNO-Lehre? Chancen und Grenzen aus Sicht von Studierenden und Lehrenden

**DOI:** 10.1007/s00106-024-01437-8

**Published:** 2024-02-19

**Authors:** Lisa Schmitz, Christian S. Betz, Arne Böttcher, Sophia M. Häußler, Mark Praetorius

**Affiliations:** 1 Klinik und Poliklinik für Hals‑, Nasen‑, Ohrenheilkunde, Universitätsklinikum Hamburg-Eppendorf, Hamburg, Deutschland; 2 Klinik und Poliklinik für Hals‑, Nasen- und Ohrenheilkunde, Kopf- und Neurozentrum, Universitätsklinikum Hamburg-Eppendorf, Martinistr. 52, 20246 Hamburg, Deutschland

**Keywords:** Digitale Technologie, Medizinstudierende, Interaktives Lernen, Motivation, Zufriedenheit, Digital technology, Medical students, Interactive learning, Motivation, Satisfaction

## Abstract

**Hintergrund:**

Digitalisierung ist längst fester Bestandteil des Alltags von Studierenden und zunehmend auch ihrer medizinischen Ausbildung. Es scheint ein ungeschriebenes Gesetz zu sein, dass „digital natives“ möglichst viel Digitalisierung wollen. In dieser Studie wurde beleuchtet, wie Studierende im klinisch-geprägten Abschnitt des Medizinstudiums die zunehmende Digitalisierung der Lehre empfinden und was sie für eine gute Ausbildung benötigen.

**Material und Methoden:**

Die vorliegende Studie analysiert 2 Umfragen, welche mittels Online-Fragebogen erhoben wurden. Zum einen wurden Studierende des 5.–9. Fachsemesters der Medizinischen Fakultät der Universität Hamburg (*n* = 282) befragt (Umfrage 1). Eine weitere Umfrage adressierte alle Beschäftigten der HNO-Kliniken Deutschlands (*n* = 175; Umfrage 2).

**Ergebnisse:**

Es nahmen 76 Studierende an Umfrage 1 und 123 Dozierende an Umfrage 2 teil. Die Ergebnisse zeigen, dass sowohl Studierende als auch Dozierende keinen vollumfänglichen Ersatz von Präsenzlehre durch digitale Formate wünschen. Insgesamt 72,7 % der Studierenden lehnen die Möglichkeit der Vermittlung praktischer Fertigkeiten durch digitale Formate ab. Der Großteil der befragten Studierenden gibt an, in Offline-Formaten eine bessere Konzentration (61,1 %), Teilnahmewahrscheinlichkeit (63,9 %) und Lernmotivation (76,6 %) zu haben. Dozierende hingegen sehen die Digitalisierung zu 40,2 % als Entlastungsmöglichkeit ohne relevante Qualitätsverluste der Lehre.

**Schlussfolgerung:**

Digitale Lehrformate beeinflussen die medizinische Ausbildung der befragten Studierenden negativ. Es bedarf der Interaktion und physischen Anwesenheit zur Steigerung der Lernmotivation. Dies führt zu dem ersten Schluss, dass Studierende einer zunehmenden Digitalisierung des Medizinstudiums kritisch gegenüberstehen.

## Abstract (en)

**Background:**

Digitalization has long been an integral part of students’ everyday lives and increasingly also of their medical training. It seems to be an unwritten law that “digital natives” want as much digitalization as possible. This study aims to shed more light on how students in the clinical phase of medical studies perceive the increasing digitalization of teaching and what they need for good education.

**Materials and methods:**

This study analyzed two surveys that were conducted using an online questionnaire. On the one hand, students in the 5th–9th semesters of the medical faculty at the University of Hamburg (*n* = 282) were surveyed (survey 1). Another survey addressed all employees of ENT clinics in Germany (*n* = 175; survey 2).

**Results:**

A total of 76 students took part in survey 1 and 123 lecturers in survey 2. The results show that both students and lecturers do not want face-to-face teaching to be completely replaced by digital formats. A total of 72.7% of students reject the possibility of teaching practical skills through digital formats. The majority of students surveyed stated that offline formats improve their concentration (61.1%), participation probability (63.9%), and motivation to learn (76.6%). In contrast, 40.2% of lecturers see digitalization as a way to reduce the workload without any relevant loss in teaching quality.

**Conclusion:**

Digital teaching formats have a negative impact on the medical education of the students surveyed. Interaction and physical presence are needed to increase the motivation to learn. This leads to the first conclusion that students are critical of the increasing digitalization of medical studies.

## Digitale Konzepte

In einer Zeit der digitalen Transformation müssen sich Bildungseinrichtungen der Herausforderung stellen, ihr didaktisches Konzept an aktuelle Bedürfnisse der Studierenden und der technologisierten Gesellschaft anzupassen. Erste digitale Konzepte in der medizinischen Lehre wurden bereits in den frühen 2000er-Jahren evaluiert, eine flächendeckende, vernetzende Verankerung digitaler Lehr- und Lernstrukturen etablierte sich jedoch nur zögerlich [9, 29, 30]. Verantwortlich hierfür waren in Deutschland v. a. finanzielle, strukturelle und administrative Hürden. Der plötzliche, übergeordnete Bedarf einer digitalen Lösung für die Fortführung der medizinischen Ausbildung aufgrund der SARS-CoV-2-Pandemie ab März 2020 führte zu einer Senkung institutioneller Hürden und wurde so zum unerwarteten Motor der Digitalisierung in der medizinischen Didaktik [4, 23].

Für die Hals‑, Nasen- und Ohrenheilkunde (HNO) wurden viele innovative Formate wie eine interaktive, digitale Patientenvorstellung [21], ein interaktives, multimodulares Blockpraktikum [7], fallbasierte interaktive Lernprogramme [26], Anatomieunterricht mit virtueller Realität [27] oder auch digitale Untersuchungskurse [8, 16] eingeführt. Während sich die Ergebnisse der Lernerfolge hier wesentlich unterschieden, zeigte sich einheitlich die Studierendenzufriedenheit als wichtiger Marker in den Erhebungen. Anfänglich werden digitale Lehrformate meist als spannend und unterhaltsam empfunden [28]. Die Akzeptanz eines umfänglichen Einsatzes digitaler Lehre ohne Interaktion oder Präsenzanteil sinkt ohne nachvollziehbare Gründe, wie z. B. eine Pandemie, jedoch schnell ab [17, 28].

Für Dozierende ist die Durchführung universitärer Lehre im Rahmen des klinischen Alltags oft eine Zusatzbelastung, die zu wenig Zeit eingeräumt bekommt. „Teaching on the run“ [11] ist dabei mittlerweile eher die Regel als die Ausnahme. Deshalb ist aus Sicht der Lehrenden die zunehmende Digitalisierung mit Etablierung von Formaten wie „flipped classroom“ oder digitalen Fallakten verständlicherweise eine attraktive Lösung. Nur so erscheint in Zeiten von Personalmangel und Leistungsdruck die Machbarkeit der medizinischen Ausbildung noch zu gewährleisten zu sein.

Die vorliegende Arbeit soll nun analysieren, wie die Akzeptanz und Bedürfnisse von Studierenden hinsichtlich der zunehmenden Digitalisierung des Medizinstudiums sind, und sie beleuchtet zudem die Einschätzung von Dozierenden der HNO in Deutschland.

## Material und Methoden

Die Datenerhebung der Studierenden (Umfrage 1) erfolgte mittels einer Online-Umfrage mit 10 Items zur Digitalisierung der Lehre, welche per Mail an alle Studierenden (*n* = 282) des 5. bis 9. Semesters mit Lehrveranstaltungen in der Hals‑, Nasen- und Ohrenheilkunde im Wintersemester 2021/22 an der Medizinischen Fakultät der Universität Hamburg versendet wurden. Die Rücklaufzeit betrug 60 Tage. Die Einschätzung erfolgte auf einer 5‑stufigen endpunktbenannten Skala von 1 = starke Ablehnung bis 5 = starke Zustimmung.

Die Datenerhebung der Lehrenden (Umfrage 2) erfolgte mittels einer Online-Umfrage mit 6 Items, welche an sämtliche Klinikdirektoren mit HNO-Abteilung in Deutschland, die auf der Homepage der Deutschen Gesellschaft für Hals‑, Nasen- und Ohrenheilkunde gelistet waren (*n* = 129), und an alle Klinikdirektoren der Universitätskliniken in Deutschland (*n* = 36) mit der Bitte um Distribution innerhalb des Kollegiums versendet wurde. Die Fragebögen wurden im Mai 2022 mit einer Rücklaufzeit von 61 Tagen versendet.

Es wurden Fragen zur Digitalisierung der Lehre generell und spezifisch zur HNO-Lehre der entsprechenden Fakultät/Institution gestellt. Die Einschätzung erfolgte ebenso auf einer 5‑stufigen endpunktbenannten Skala von 1 = starke Ablehnung bis zu 5 = starke Zustimmung. Zur Wahrung der Anonymität wurde auf eine Erfassung des Standorts verzichtet.

Ein Vergleich der beiden Umfragen (1 und 2) erfolgte nicht, da es sich um unterschiedliche Populationen handelt.

Die deskriptive Auswertung der Ergebnisse erfolgte mittels SPSS (IBM SPSS Statistics für Mac, Version 27 Fa. IBM Corp., Armonk, NY, USA). Die Erstellung der Tabellen erfolgte mit Excel und PowerPoint (Fa. Microsoft, Redmond, WA, USA).

Da diese Umfrage eine rein anonyme Erhebung und Nutzung von Daten darstellt, bei der zu keinem Zeitpunkt eine Schlüsselliste bestand und an der alle Studierenden und Ärzt:innen freiwillig teilnahmen, zur Einholung besteht keine Beratungszuständigkeit nach § 15 der Berufsordnung und keine Notwendigkeit eines Ethikvotums. Die ethischen Standards der Medizinischen Fakultät Hamburg wurden gemäß der aktuell gültigen Helsinki-Deklaration berücksichtigt. Es bestehen keinerlei Interessenkonflikte.

## Ergebnisse

### Studierendenbefragung

Von 76 der 282 angeschriebenen Studierenden kam eine vollständig ausgefüllte Rückmeldung (Rücklaufquote von 26,9 %). Insgesamt waren 64 % der Teilnehmenden weiblich, 35 % männlich, und eine Person identifizierte sich als nonbinär. Das Alter der Befragten lag im Mittel bei 25,7 Jahren (Spannbreite: 21–46 Jahre). Von den Befragten gaben 23,4 % an, dass die HNO für sie als Weiterbildungsfach in Betracht komme, jedoch gaben nur 2,6 % aller Befragten an, dass die HNO ihr aktueller Fachweiterbildungswunsch sei.

**Figure 1:**
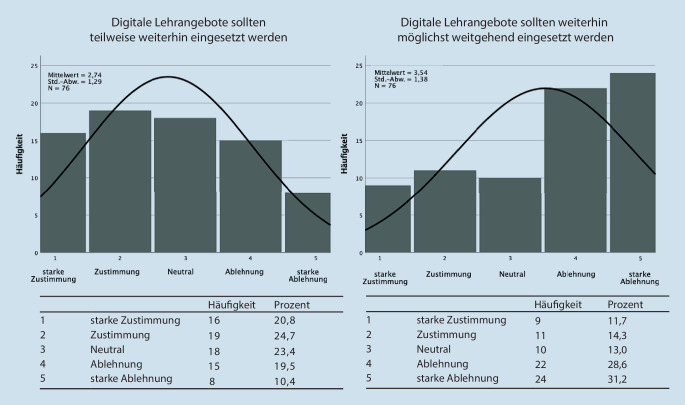

Im ersten Abschnitt der Befragung wurde die zukünftig gewünschte Quantität digitaler Lehrformate in der HNO-Lehre erfasst (Abb. 1). Die befragten Studierenden stimmten zu 45,5 % zu, dass digitale Lehre zumindest teilweise in der HNO weiter eingesetzt werden sollte. Knapp die Hälfte davon zeigte sogar eine starke Zustimmung. Einen möglichst weitreichenden Einsatz digitaler Lehre lehnten jedoch 59,8 % der Studierenden ab.

**Figure 2:**
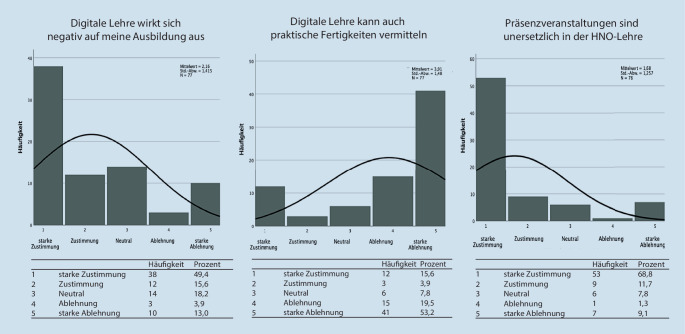

Die subjektiv empfundene Auswirkung digitalisierter Lehrformate auf ihre Ausbildung wurde im zweiten Abschnitt der Befragung erfasst (Abb. 2). Hier stimmten 49,4 % der Befragten einem negativen Einfluss digitaler Lehre auf ihre Ausbildung stark zu. Nur 16,9 % empfanden keine negative Beeinflussung digitaler Lehre auf ihre Ausbildung. Die Möglichkeit der Vermittlung praktischer Kompetenzen durch digitale Lehre lehnten 72,7 % der Studierenden ab, der Großteil gab hierbei eine starke Ablehnung an. Ergänzend dazu stimmten 68,8 % der Studierenden der Hypothese stark zu, dass Präsenzveranstaltungen in der Lehre der HNO unersetzlich seien.

**Figure 3:**
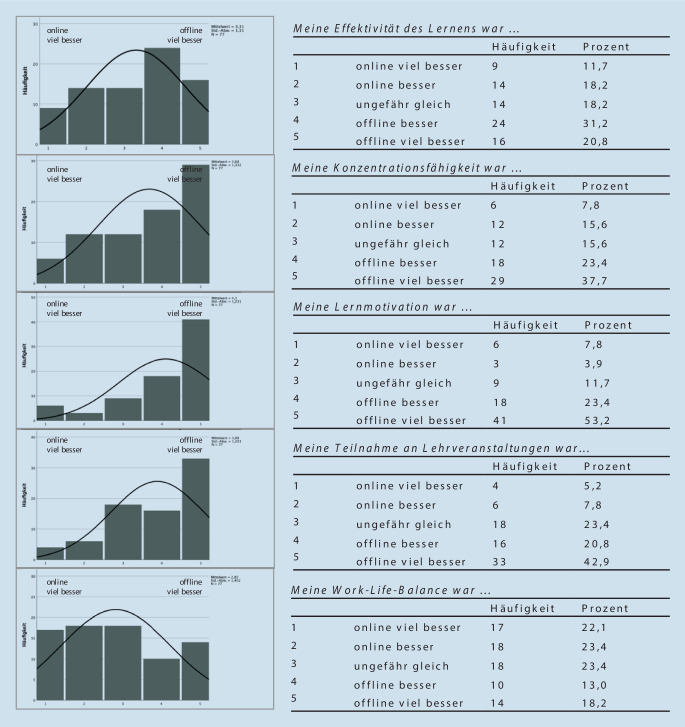

Im dritten Item-Abschnitt wurde die Einschätzung der Studierenden hinsichtlich des Einflusses von digitaler gegenüber Präsenzlehre (Offline-Lehre) auf verschiedene Parameter der Lernumgebung beleuchtet (Abb. 3). Bei der Effektivität des Lernens gaben 52 % der Studierenden an, dass diese in Offline-Veranstaltungen besser sei, immerhin 29,9 % gaben dahingegen an, diese sei in Online-Formaten besser. Insgesamt 61,1 % der Studierenden stimmten zu, dass in Offline-Lehrformaten ihre Konzentrationsfähigkeit besser sei. Etwa genauso viele Studierende (63,8 %) stimmten zu, dass ihre Teilnahmewahrscheinlichkeit an Offline-Lehrveranstaltungen besser sei als an Online-Formaten. Dabei gaben 42,9 % der Befragten sogar eine viel bessere Teilnahme an. Bei der Lernmotivation gaben 76,6 % aller befragten Studierenden an, dass diese in Offline-Formaten besser sei, 53,2 % davon gaben eine viel bessere Motivation an.

### Dozierendenbefragung

Von den insgesamt 175 adressierten deutschen HNO-Kliniken wurde von 107 Personen der Rücklauf eines vollständig ausgefüllten Fragebogens verzeichnet. Insgesamt waren 46 % der Teilnehmenden weiblich und 54 % männlich, das durchschnittliche Alter lag bei 38,8 Jahren (Spannbreite: 25–64 Jahre), und 62 % waren zum Zeitpunkt der Befragung an einem Universitätsklinikum beschäftigt. Insgesamt waren 38 % der teilnehmenden Assistenzärzt:innen in Weiterbildung, 17 % Fachärzt:innen und 45 % Ober- oder Chefärzt:innen.

**Figure 4:**
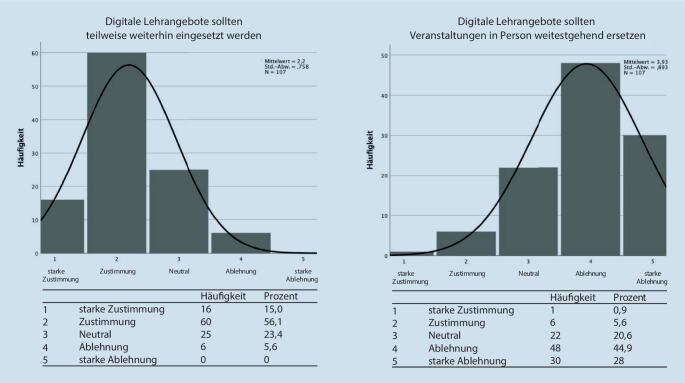

Im ersten Abschnitt der Befragung wurde auch hier die zukünftig gewünschte Quantität digitaler Lehrformate in der HNO-Lehre erfasst (Abb. 4). Dabei stimmten 71,1 % der Befragten zu, dass digitale Lehrangebote weiterhin teilweise eingesetzt werden sollen. Einen weitgehenden Einsatz digitaler Lehre lehnten 72,9 % ab (44,9 % Ablehnung +28 % starke Ablehnung). Für einen weitestgehenden Ersatz von Präsenzlehre sprachen sich nur 6,5 % der befragten Dozierenden aus.

**Figure 5:**
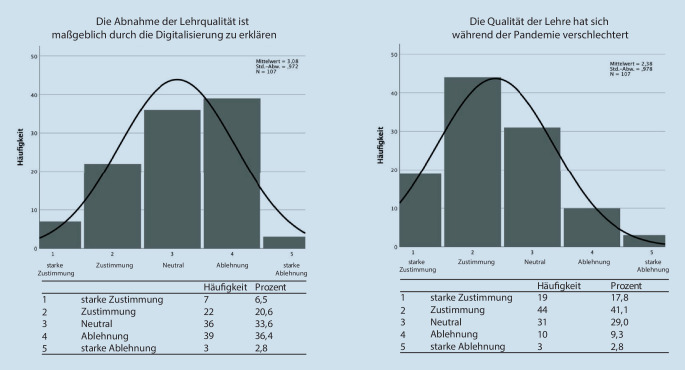

Im zweiten Abschnitt der Befragung wurde die subjektiv empfundene Qualität der Lehre während der SARS-CoV-2-Pandemie erfasst (Abb. 5). Während sich 58,9 % der Befragten einig waren, dass die Lehrqualität in den Jahren der Pandemie gesunken sei, waren nur 27,1 % der Befragten der Meinung, die Digitalisierung habe einen maßgeblichen Anteil daran. Diese Hypothese wurde von 39,2 % der Befragten abgelehnt.

**Figure 6:**
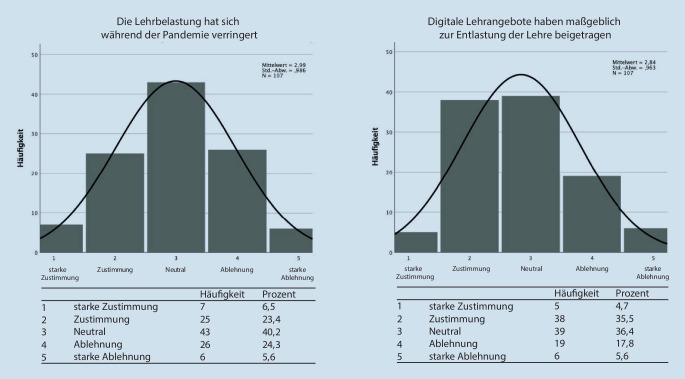

Im dritten Abschnitt der Befragung wurde die empfundene Lehrbelastung der Dozierenden erfragt (Abb. 6). Dabei stimmten 29,9 % einer subjektiv empfundenen Verringerung der Lehrbelastung während Pandemie zu, genauso viele (29,9 %) lehnten diese Aussage ab. Insgesamt gaben jedoch 40,2 % der Befragten an, dass die Digitalisierung maßgeblich zu einer Entlastung in der Lehre beigetragen habe. Nur 23,4 % der Befragten verneinten dies.

## Diskussion

Die Ergebnisse der vorliegenden Befragungen von Studierenden sowie Dozierenden hinsichtlich des Einsatzes digitaler Lehre in der HNO legen nahe, dass das Ausmaß der Digitalisierung insbesondere aus der Studierendenperspektive kritisch zu evaluieren ist.

Wie in den Abb. 1 und 4 dargestellt, ist sich der Großteil aller Befragten Studierenden und Dozierenden einig, dass Präsenzveranstaltungen nicht gänzlich ersetzt werden sollten.

In Umfrage 1 lehnten knapp 2 Drittel der Studierenden einen weitreichenden Einsatz digitaler Lehre ab. Dies scheint insbesondere mit der Einschätzung verknüpft zu sein, dass sich digitale Lehrformate negativ auf ihre Ausbildung auswirken würden. Auch wenn hier der Lernerfolg nicht explizit wörtlich abgebildet wird, liegt der Schluss nahe, dass ein negativer Einfluss auf die Ausbildung mit einem geringer eingeschätzten Lernerfolg verknüpft ist. Zukünftige Befragungen hierzu müssen zeigen, ob sich diese Hypothese bestätigt.

In der Literatur finden sich inhomogene Ergebnisse im Vergleich zwischen Online- und Offline-Formaten [1, 2, 10, 19, 20]. Metaanalysen von Liu et al. und Lockey et al. konnten jedoch zeigen, dass Hybridformate wie „blended learning“ hinsichtlich des Wissenszuwachses zumindest vergleichbar mit analogen Formaten sein können [13, 14]. Dies unterstreicht die Vermutung der Autoren, dass sich ein essenzieller Teil des negativen Einflusses besonders in der Vermittlung praktischer Inhalte wiederfindet. Denn ein Großteil der befragten Studierenden gab an, dass das Erlernen praktischer Fertigkeiten nicht durch digitale Formate abgebildet werden könne. Auch in anderen Studien, die während der Pandemie durchgeführt wurden und aufwendige digitale Lehrkonzepte wie ein interaktives Blockpraktikum evaluierten, wurde gezeigt, dass trotz verbesserter Evaluationen v. a. der fehlende Patientenkontakt von Studierenden stark kritisiert wurde [7, 18, 21]. Diese Ansicht wird durch die Studie von Lüdke et al. untermauert, in der die Autoren den Lernerfolg einer rein digital vermittelten Kopf-Hals-Untersuchung in der HNO ohne Interaktion mit Lehrenden untersuchten und feststellen mussten, dass die Ergebnisse denen aus der Präsenzlehre unterlegen sind [16]. Jedoch konnten sowohl Lüdke et al. als auch Lang et al. zeigen, dass ergänzende interaktive Einheiten mit Dozierenden während einer Unterrichtseinheit den Lernerfolg insgesamt signifikant unterstützten und bei Studierenden auch zu einer hohen Zufriedenheit und Motivation sowie gesteigertem Interesse am Fach HNO führten [12]. Dies weist darauf hin, dass ein essenzieller Teil in der Wissensvermittlung v. a. die Möglichkeit zur Interaktion, zum Feedback und zum Diskurs zwischen Studierenden und Lehrenden ist und somit synchrone Lehrveranstaltungen besser geeignet sind.

Auch die vorliegende Studie unterstreicht die Relevanz von Dozierendenkontakt sowie Interaktion zwischen ihnen und Studierenden und lässt eine Überlegenheit der Präsenzlehre vermuten. Eine Limitation dieser Studie besteht darin, dass das Wort Online-Lehre vorher nicht klar als synchrones oder asynchrones Format definiert wurde. Da jedoch synchrone Lehrformate den Hauptteil der Lehreinheiten der Fakultät der Autoren während der Pandemie darstellten, ist davon auszugehen, dass dies von den Studierenden dementsprechend interpretiert wurde. Nichtsdestotrotz scheint für einen Großteil der Studierenden der Unterricht in Präsenz einen höheren Stellenwert zu haben. Daraus lässt sich ableiten, dass neben der Interaktion zwischen Dozierenden und Studierenden ein weiterer wesentlicher Faktor die veränderte Lernumgebung darstellt. Denn sowohl die Lerneffektivität als auch die Konzentrationsfähigkeit wurden von den befragten Studierenden in Offline-Formaten (Präsenz-Lehre) als besser angegeben im Vergleich zur Online-Lehre. Diese beiden Surrogatparameter sind essenzielle Werkzeuge für die Unterstützung des Lernerfolgs. Darüber hinaus zeichnet sich v. a. ab, dass die befragten Studierenden mit einer höheren Wahrscheinlichkeit an einer Offline-Veranstaltung teilnehmen würden und Präsenzlehre die Lernmotivation steigere. Anhand dieser Ergebnisse lässt sich schließen, dass die Lernumgebung von Offline-Formaten für „digital natives“ viele positive Einflüsse zu haben scheint, die ihnen digitale Lehrformate nicht bieten können. Dies mag v. a. daran liegen, dass die Digitalisierung insbesondere das Aufmerksamkeitsvermögen, aber auch die Gedächtnisleistung negativ beeinflusst. Besonders das hohe Ablenkungspotenzial im digitalen Raum ist hier maßgeblich beteiligt [15]. Die Ergebnisse von Lodge et al. legen nahe, dass die Befragung auch ohne die negativen Einflüsse der Corona-Pandemie auf die Studierenden ähnlich hätte ausfallen können. Es scheint somit logisch, dass in einer zunehmend technologisierten Welt ein reeller Kontakt im klar örtlich und zeitlich definierten Raum für Studierende ganz abseits von Lernerfolg und medizinischer Ausbildung als sehr wertvoll erachtet wird und aus diesem Grund unersetzlich erscheint.

In Umfrage 2 der Dozierenden wurde die subjektiv empfundene Qualität der Lehre während der ausnahmslos digital stattfindenden Lehrperiode im Rahmen der Corona-Pandemie erfasst. Ein Großteil der Befragten gab hier zwar eine gesunkene Qualität an, jedoch beurteilte nur ein Viertel die Digitalisierung der Lehre als maßgeblich ursächlich für diesen Qualitätsverlust. Hier scheinen insbesondere zeitliche und personelle Ressourcenknappheit eine entscheidende Rolle in der Einschätzung gespielt zu haben. Dann stellt sich natürlich aber die Frage, ob diese Wahrnehmung der Dozierenden eine Fehleinschätzung ist, da die befragten Studierendenden eindeutig eine schlechtere Lehrqualität in den Online-Veranstaltungen angaben. Die Erstellung einer direkten statistischen Korrelation ist in diesem Falle jedoch nicht möglich, da die Populationen nicht dieselben Unterrichtseinheiten beurteilen.

Wesentlich in der Befragung der Dozierenden erscheint v. a. der dritte Abschnitt der Befragung. Weniger als ein Drittel (29,9 %) der Befragten gaben eine Reduktion der Lehrbelastung an. Jedoch sprachen sich über 40 % dafür aus, dass digitale Lehrangebote maßgeblich zu einer Entlastung beigetragen hätten. Dies legt den Schluss nahe, dass auch wenn Dozierende keine persönliche Entlastung in der Lehre während der Pandemie empfunden haben, diese trotzdem übergeordnet eine Entlastung durch digitale Formate in der Lehre wahrnehmen.

Insgesamt bieten digitale Lehr- und Lernstrukturen für Studierende und Dozierende trotzdem viele Vorteile [5, 22, 25]. Insbesondere die starke Individualisierbarkeit von Lernmaterial und Lernmethoden, beispielsweise mit „adaptive e‑learning environments“ [3, 5] und „gamification“ [24], könnten hilfreiche Werkzeuge in der Optimierung der Lernerfahrung sein. Das bestehende Bedürfnis der „digital natives“ nach Interaktion, Patientenkontakt und einem physischen Raum im Rahmen der medizinischen Ausbildung wird jedoch auch in Zukunft zumindest in Teilen in Präsenz abgebildet werden müssen. Ob Hybridkonzepte wie ein „flipped classroom“ diese Bedürfnisse umfänglich befriedigen können, müssen zukünftige größer angelegte Studien erheben. Und auch eine Korrelation mit objektivierbaren Parametern wie quantitativer Lernerfolg ist im Rahmen dieser Erhebung qualitativer Werte nicht erfolgt. Hier können zukünftige Studien das Bild weiter vervollständigen.

Relevant bleibt darüber hinaus auch der Aspekt der personellen Verfügbarkeit und damit einhergehend die Lehrbelastung der Dozierenden. Denn nur langfristige und bestenfalls standortübergreifende Projektimplementierungen können eine reelle Personalentlastung erzielen. Mögliche Lösungen sehen die Autoren hier in der Nutzung und Erweiterung von „open educational ressources“ (frei online verfügbare und nutzbare Lehrmaterialien zur optimierten Ressourcennutzung) [6].

## Fazit für die Praxis

- In Zukunft wird die Herausforderung für die medizinische Lehre v. a. darin bestehen, im Spannungsfeld zwischen divergierenden Bedürfnissen weiterhin einen definierten Rahmen für Studierende zur Verfügung zu stellen und gleichzeitig auch die Bedürfnisse der Dozierenden angesichts einer stetig steigenden Arbeitsbelastung zu berücksichtigen.
- Hierzu müssen weitere Studien durchgeführt werden, die verschiedene Formate hinsichtlich Lernumgebungen, Lernerfolg und Lehrbelastung näher beleuchten.

## References

[1] Chen F, Lui AM, Martinelli SM (2017). A systematic review of the effectiveness of flipped classrooms in medical education. Med Educ.

[2] Dombrowski T, Wrobel C, Dazert S (2018). Flipped classroom frameworks improve efficacy in undergraduate practical courses—a quasi-randomized pilot study in otorhinolaryngology. BMC Med Educ.

[3] Ellaway RH, Pusic MV, Galbraith RM (2014). Developing the role of big data and analytics in health professional education. Med Teach.

[4] Fischer MR (2021). Digital teaching after the pandemic—enriching diversity of teaching methods and freedom for inclination-oriented learning?. GMS J Med Educ.

[5] Fontaine G, Cossette S, Maheu-Cadotte MA (2019). Efficacy of adaptive e-learning for health professionals and students: a systematic review and meta-analysis. BMJ Open.

[6] Khalid F, Wu M, Ting DK (2023). Guidelines: the do’s, don’ts and don’t knows of creating open educational resources. Perspect Med Educ.

[7] Krambeck A, Loth AG, Leinung M (2022). Hat die SARS-CoV-2-Pandemie die Lehre verbessert? – Virtueller Unterricht im Fach HNO-Heilkunde aus Sicht der Studierenden. HNO.

[8] Krauss F, Giesler M, Offergeld C (2022). On the effectiveness of digital teaching of practical skills in curricular ENT education. HNO.

[9] Kuhn S, Frankenhauser S, Tolks D (2018). Digital learning and teaching in medical education : already there or still at the beginning?. Bundesgesundheitsblatt Gesundheitsforschung Gesundheitsschutz.

[10] Kyaw BM, Posadzki P, Paddock S (2019). Effectiveness of digital education on communication skills among medical students: systematic review and meta-analysis by the digital health education collaboration. J Med Internet Res.

[11] Lake FR (2004). Teaching on the run tips: doctors as teachers. Med J Aust.

[12] Lang F, Everad B, Knopf A (2021). Digitalization in curricular teaching: experiences with the Freiburg ENT learning program. Laryngorhinootologie.

[13] Liu Q, Peng W, Zhang F (2016). The effectiveness of blended learning in health professions: systematic review and meta-analysis. J Med Internet Res.

[14] Lockey A, Bland A, Stephenson J (2022). Blended learning in health care education: an overview and overarching meta-analysis of systematic reviews. J Contin Educ Health Prof.

[15] Lodge JM, Harrison WJ (2019). The role of attention in learning in the digital age. Yale J Biol Med.

[16] Ludke T, Polk ML, Gunther S (2023). Digital teaching and assessment of psychomotor skills of the clinical head and neck examination during COVID-19 pandemic. Eur Arch Otorhinolaryngol.

[17] Mustafa S, Qiao Y, Yan X (2022). Digital students’ satisfaction with and intention to use online teaching modes, role of big five personality traits. Front Psychol.

[18] Olmes GL, Zimmermann JSM, Stotz L (2021). Students’ attitudes toward digital learning during the COVID-19 pandemic: a survey conducted following an online course in gynecology and obstetrics. Arch Gynecol Obstet.

[19] Raupach T, Grefe C, Brown J (2015). Moving knowledge acquisition from the lecture hall to the student home: a prospective intervention study. J Med Internet Res.

[20] Sadeghi R, Sedaghat MM, Ahmadi SF (2014). Comparison of the effect of lecture and blended teaching methods on students’ learning and satisfaction. J Adv Med Educ Prof.

[21] Seiwerth I, Bartel S, Herzog M (2022). Ausbildung in COVID-19-Pandemie-Zeiten: Wie bewerten Medizinstudierende einen interaktiven, videobasierten Distanzunterricht am Patienten im Fach Hals-Nasen-Ohren-Heilkunde?. HNO.

[22] Stegmann K, Fischer F (2016). Auswirkungen digitaler Medien auf den Wissens- und Kompetenzerwerb an der Hochschule.

[23] Tolks D, Kuhn S, Kaap-Frohlich S (2020). Teaching in times of COVID-19. Challenges and opportunities for digital teaching. GMS J Med Educ.

[24] van Gaalen AEJ, Brouwer J, Schonrock-Adema J (2021). Gamification of health professions education: a systematic review. Adv Health Sci Educ Theory Pract.

[25] Vaona A, Banzi R, Kwag KH (2018). E-learning for health professionals. Cochrane Database Syst Rev.

[26] Vielsmeier V, Auerswald S, Marienhagen J (2020). Digital teaching with interactive case presentations of ENT diseases—discussion of utilisation and motivation of students. GMS J Med Educ.

[27] von Schnakenburg P, Heermann S, Kromeier J (2023). Use of virtual reality in ENT teaching: an alternative to the conventional anatomic model. HNO.

[28] Warnecke E, Pearson S (2011). Medical students’ perceptions of using e-learning to enhance the acquisition of consulting skills. Australas Med J.

[29] Wissing F (2020). Digitale Lehre für alle: Voraussetzungen, Machbarkeit und Optionen im Human- und Zahnmedizinstudium Hintergrund.

[30] Yeung AWK, Parvanov ED, Hribersek M (2022). Digital teaching in medical education: scientific literature landscape review. JMIR Med Educ.

